# Measuring Biomechanical Risk in Lifting Load Tasks Through Wearable System and Machine-Learning Approach

**DOI:** 10.3390/s20061557

**Published:** 2020-03-11

**Authors:** Ilaria Conforti, Ilaria Mileti, Zaccaria Del Prete, Eduardo Palermo

**Affiliations:** Department of Mechanical and Aerospace Engineering, Sapienza University of Rome, 00184 Rome, Italy; ilaria.mileti@uniroma1.it (I.M.); zaccaria.delprete@uniroma1.it (Z.D.P.)

**Keywords:** activity recognition, wearable sensors, risk assessment, musculoskeletal disorders, motion analysis, working activities, machine-learning algorithms

## Abstract

Ergonomics evaluation through measurements of biomechanical parameters in real time has a great potential in reducing non-fatal occupational injuries, such as work-related musculoskeletal disorders. Assuming a correct posture guarantees the avoidance of high stress on the back and on the lower extremities, while an incorrect posture increases spinal stress. Here, we propose a solution for the recognition of postural patterns through wearable sensors and machine-learning algorithms fed with kinematic data. Twenty-six healthy subjects equipped with eight wireless inertial measurement units (IMUs) performed manual material handling tasks, such as lifting and releasing small loads, with two postural patterns: correctly and incorrectly. Measurements of kinematic parameters, such as the range of motion of lower limb and lumbosacral joints, along with the displacement of the trunk with respect to the pelvis, were estimated from IMU measurements through a biomechanical model. Statistical differences were found for all kinematic parameters between the correct and the incorrect postures (*p* < 0.01). Moreover, with the weight increase of load in the lifting task, changes in hip and trunk kinematics were observed (*p* < 0.01). To automatically identify the two postures, a supervised machine-learning algorithm, a support vector machine, was trained, and an accuracy of 99.4% (specificity of 100%) was reached by using the measurements of all kinematic parameters as features. Meanwhile, an accuracy of 76.9% (specificity of 76.9%) was reached by using the measurements of kinematic parameters related to the trunk body segment.

## 1. Introduction

The assumption of non-neutral postures during working activities is the main cause of injuries and accidents in a workplace [1,2,3,4,5,6]. Manual material handing (MMH) tasks expose workers to a high level of ergonomic hazards, which finds high correlation with the onset of work-related musculoskeletal disorders (WMSDs) [7,8,9,10,11]. Awkward postures have been identified as risk factors for the musculoskeletal system, especially in the construction field [12,13,14,15]. Though different guidelines have been designed for risk assessments, they have not always been definitive due to factors such as high variability, repetitiveness, and a low standardization of tasks [16,17,18,19,20,21,22]. Ergonomics investigations, based on measurements of kinematic and dynamic parameters that are obtained through motion analysis, might be helpful in designing risk management in a real manufacturing scenario.

Construction workers perform activities that demand the repetitive lifting of loads with different weights, the carrying of heavy loads, or being in a posture that is difficult to standardize [3,23,24]. For instance, when considering MMH tasks such as lifting loads (LL), lower back pain, and lower limb joints are the effect of WMSDs due to the adoption of incorrect postural patterns in this kind of activity. According to guidelines, reducing the occurrence of musculoskeletal risks in LL tasks goes through: (i) maintaining a straight spine, (ii) avoiding the full flexion of the trunk for a long period, (iii) leveraging the lower extremities during the lifting phase, and (iv) bringing the load close to the body in order to reduce the distance between the center of mass of the object and the center of the mass of the human body [23]. Furthermore, it is essential to use better motion control through safer posture, which limits, or helps to compensate for, a non-neutral distribution of biomechanical loads on the back and the lower extremities. In 2003, Leon Straker compared three different techniques for lifting low-lying objects: the squat, stoop, and semi-squat techniques [25]. The squat technique was highly supported by many researches after observing high values of compression, shear force and peak lumbar moments in the stoop technique [25,26,27].

Observational-based methods have usually been adopted to evaluate working posture and fatigue level [28,29,30,31,32]. Though they are easy to apply to working places, they are biased by the subjectivity of the raters. There is a need for objective quantification-fueled research efforts involving instrumentation for motion analysis. Hwang et al. investigated the lifting squat and stoop techniques by estimating kinematic and kinetic parameters through motion capturing solutions such as an optoelectronic system (OS) and force plates [33]. These two devices are known for their accuracy and reliability on measurements of kinematic variables, but they are difficult to introduce in a workplace setting [11,22,33,34,35]. Continuous monitoring of a working site could be obtained through the adoption of wearable motion capture systems that are suitable for outdoor motion analysis [2,4,9,10,17,36,37,38,39]. Wearable devices, like inertial measurement units (IMUs), represent the most suitable technologic solution for gathering reliable measurements of kinematic parameters and performing motion analysis in a real manufacturing scenario. In clinics, sports, daily life activities, and industrial applications, the adoption of IMUs has enabled promising results [40,41,42,43]. 

Estimating biomechanical risks in MMH tasks through IMUs [4,37,39] allows for the design of setups that are not bulky and that suitable for each working activity. In 2016, Gholipour and colleagues assessed spinal posture during lifting tasks through three inertial sensors placed on pelvis and trunk segments [38]. In 2017, Yan and colleagues designed a small setup to analyze the working postures of construction workers in real time [4]. The setup was composed of two IMUs placed on the head and trunk, preventing the detection of WMSDs related to the upper body, such as neck, shoulder and low back pain [4,39]. Nath et al. focused on assessing the ergonomic risk levels of awkward postures only through sensor data related to trunk and shoulder segments [9]. Even if these small setups do not hamper workers in their MMH activities, they do not provide any information about lower limb kinematics, which has been demonstrated to be highly involved in lifting tasks [33]. In fact, in our previous work, a dependency on the weight of kinematic parameters related to the lower limbs during the manual handling of small loads was found. This result highlighted the necessity to assess lower extremity motion despite the use of small setups, especially in the real-time evaluation of postural patterns [43].

The integration of a real-time posture analysis through wearables devices with machine learning algorithms could represent a useful tool to reduce WMDSs. The reliability and precision of devices that are employed for monitoring workers in their own workplace, along with the automatic detection of motion activities, might provide a requalification of the work environment. The machine learning algorithm allows for an automatic recognition of dangerous situations in several fields, such as rehabilitation and ergonomics [44,45,46,47,48,49], as well as the improvement of the adaptability of prosthetic lower limbs, as in a study by study Sun et al. [50]. Thus, hazard lifting and fall risk could be immediately identified, thus increasing the safety on working places [1,38]. In 2019, Antwi-Afari and colleagues evaluated working activity recognition through four supervised machine-learning classifiers that were fed with wearable insole pressure data [51]. Regarding posture recognition in lifting activities, Gholipour et al. investigated the ability of artificial neuronal networks (ANNs) in predicting spinal posture during lifting and reaching a 5 kg load at different heights [38].

As supervised machine-learning algorithm, the support vector machine (SVM) guarantees good outcomes classifying human motion patterns [44,47]. In industrial environments, SVMs have been applied to classify and recognize the ergonomic posture of movement patterns [40]. In the last few years, activity recognition has usually been based on machine learning algorithms that are fed with inertial sensor data [38,52]. Alwasel et al. proposed and validated an SVM algorithm that classified masonry workers’ poses as a function of their experience, obtaining a high rate of accuracy 92.11% [52]. In 2016, Ryu and colleagues tested the action recognition of masonry workers by using supervised learning algorithms, such as an SVM, a k-nearest neighbor (kNN) algorithm, and a neural network [53]. Their classifiers were fed with accelerometer data of a sensor that was only placed on an upper segment of each subject (e.g., the dominant hand) and reached an accuracy ranging between 80% and 100%. However, providing reliable worker activity recognition needs data gathering that is not limited to the upper segments. The aim of our study was to design an experimental protocol for assessing postural patterns, defining a correct and incorrect posture according to the current safety and ergonomic guidelines, and evaluating motion analysis through a wearable system and a biomechanical model. An additional aim was to test the classification potential of an SVM for recognizing the two correct and incorrect postural patterns during MMH tasks through the measurements of kinematic parameters related to lower limb, pelvis and trunk body segments. The SVM was fed with two datasets: the trunk–lower limb (TL-l) dataset, which was composed of data that were collected through eight sensors and that were related to the kinematic parameters of the trunk and the lower limbs, and the upper body (UpB) dataset, which was composed of data that were collected through two sensors that were placed on pelvis and trunk body segments. The two datasets were analyzed to unveil the most effective choice to assess human motion through a small setup in a workplace.

## 2. Materials and Methods

### 2.1. Experimental Setup

**Figure 1:**
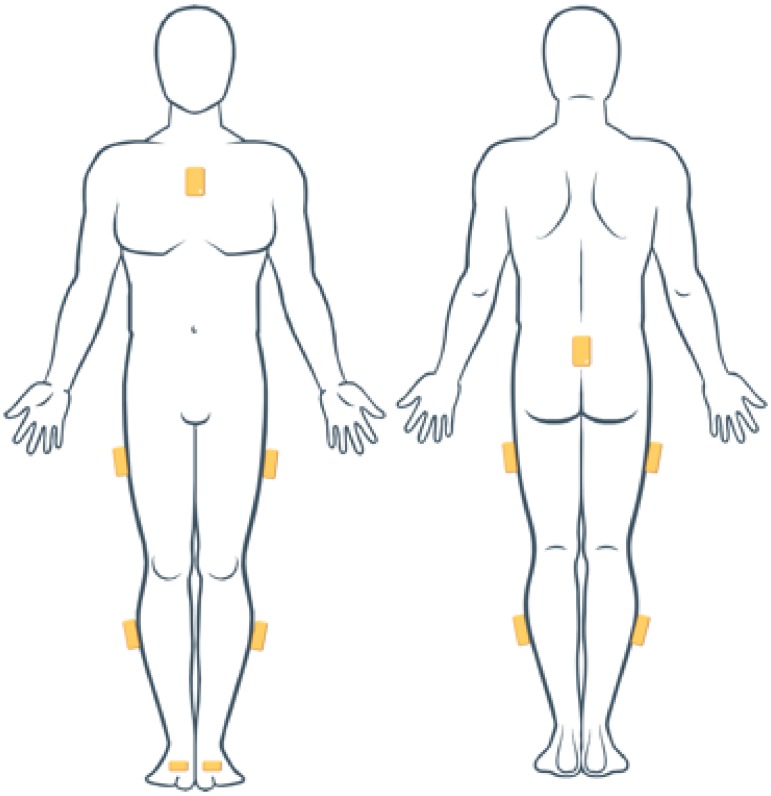
Inertial measurement units (IMUs) positions on the human body segments.

Twenty-six healthy subjects (age: 26.9 ± 4.7 years; height: 171.0 ± 7.8 cm; weight: 64.8 ± 11.8 kg) were recruited to participate in the study. Eight wireless inertial measurement units (MIMUs MTw, Xsens Technologies—NL) were used to gather kinematic data of the upper and lower body segments of each subject. More specifically, for the upper body segments, two sensors were placed (i) under the suprasternal notch on the sternum body and (ii) posteriorly on pelvis; for the lower body segments, six sensors were placed (i) laterally on the mid-thighs, (ii) laterally on the mid-shanks, and (iii) on the instep of the feet. To limit relative movements between each sensor and its body segment, each IMU was fixed with elastic straps (see Figure 1). The sampling rate was set at 40 Hz. All data were low-pass filtered by applying a second-order recursive Butterworth filter at a cutoff frequency of 10 Hz, as the dynamics of the motion did not exceed 5 Hz.

### 2.2. Experimental Procedure

The experimental protocol consisted of performing four MMH tasks—the lifting load task (LL) in (i) the correct (CLL) and (ii) incorrect (ILL) postures and the releasing load task (RL) in (iii) the correct (CRL) and (iv) incorrect (IRL) postures. Specifically, we assumed LL and RL as performed in the correct posture, when completed, maintained the trunk as straight as possible and the knee as flexed, i.e., squat lifting [54,55], according to the guidelines [23,24]. On the contrary, the incorrect posture comprised knees being fully extended and the trunk being completely bended, i.e., stoop lifting [16,24].

The subjects performed the MMH tasks with three different loads: 1, 2, and 5 kg. The execution in the correct and incorrect postures of tasks was randomized. Each combination of conditions (execution and load) was repeated three times.

Before the session, subjects were asked to perform the functional calibration procedure (FC) to assess their body-to-sensors alignment [56]. In the FC procedure, each subject kept a standing upright posture and a sitting position for five seconds. Subjects stood in a standing upright posture before MMH tasks. In the LL task, they lifted the load from the ground and returned to the previous posture. They kept the load close to the body at the elbow height before starting the RL task. In the RL task, each subject released the load from the elbow height, repositioning it onto the ground and returning to a standing upright posture with their hands along their sides. In the correct posture, subjects were advised to reduce biomechanical loads on their lower limb joints, e.g., preventing torque at ankles, maintaining heel contact with the ground, and limiting anterior knees movement over the toes. [55]. Moreover, to reduce biomechanical loads on the trunk body segment, they were asked to avoid twisting while performing the MMH tasks of the experimental protocol. The loads were placed near the feet of each subject in a box with two handles in order to guarantee a safe and comfortable grip. The box was made of plastic and had dimensions of 18 cm × 29 cm × 14 cm (height × width × depth). Subjects were asked to wear comfortable clothes and shoes without heels or any arch support. The speed for executing the tasks was self-selected. Subjects rested for two minutes between tasks to avoid fatigue effects.

### 2.3. Data Processing

All data were analyzed in post-processing by using the MATLAB (v.2018, Math Works, Natick, MA) program. The displacement in the 3D space between the pelvis and trunk body segments (*^pl^*PT*_trk_*), expressed in mm, was calculated. Moreover, the range of motion (RoM) of the lumbosacral (^RoM^Sacr), left and right hip (^RoM^Hip_L,_^RoM^Hip_R_), left and right knee (^RoM^Knee_L,_^RoM^Knee_R_), and left and right ankle (^RoM^Ankle_L,_^RoM^Ankle_R_) joint angles in the sagittal plane, expressed in degrees, were analyzed. Kinematics parameters were estimated through a biomechanical model (BM) according to [56,57]. More specifically, this biomechanical model defined a reference body frame for each body segment as follows: the *y*-axis directed along the direction of progression, the *z*-axis vertically directed and pointing upward, and the *yz*-plane parallel to sagittal plane. Joint angles were obtained by considering the rotation matrix between two adjacent body segments following the FC procedure [56]. To assess the *^pl^*PT*_trk_* vector, the anthropometric measurement of the distance between the midpoint of the anterior superior iliac spines and the sternum, approximately under the suprasternal notch, a distance denoted as l*_trk_*, was included in the BM as follows:

(1) $${}^{pl}o_{pl,}{}_{trk}=\left[\begin{array}{c} 0\\ 0\\ l_{trk} \end{array}\right]$$

Considering the anthropometric measurement of trunk l*_trk_*, *^pl^*o*_pl,trk_* is the origin of the trunk coordinate system (CS*_trk_*) with respect to the pelvis coordinate system (CS*_pl_*).

(2) $${}^{pl}T_{trk}=\left[\begin{array}{cc} {}^{pl}R_{trk} & {}^{pl}o_{pl,trk}\\ \begin{array}{ccc} 0 & 0 & 0 \end{array} & 1 \end{array}\right]$$

where *^pl^*R*_trk_* is the rotation matrix expressing the relative rotation between the pelvis body segment and the trunk body segment. Considering *^pl^*R*_trk_* and *^pl^*o*_pl,trk_*, *^pl^*T*_trk_* is the roto-translation matrix that is used to estimate the pose of the trunk body segment with respect to the pelvis body segment.

(3) $${}^{pl}T_{trk}{}^{-1}=\left[\begin{array}{cc} {}^{pl}R_{trk}{}^{T} & {-}^{{}^{pl}}R_{trk}{}^{Tpl}o_{pl,trk}\\ \begin{array}{ccc} 0 & 0 & 0 \end{array} & 1 \end{array}\right]$$

Considering the inverse of roto-translation matrix *^pl^*T*_trk_*, *^trk^*o*_trk,pl_* was obtained through this product:

(4) $${}^{trk}o_{trk,pl}={-}^{{}^{pl}}R_{trk}{}^{Tpl}o_{trk,pl}$$

(5) $${}^{pl}PT_{trk}{=}^{{}^{pl}}o_{pl,trk}{-}^{{}^{trk}}o_{trk,pl}$$

where the *^pl^*PT*_trk_* vector represents the displacement of the trunk body segment with respect to the pelvis body segment.

### 2.4. Statistical Analysis

To assess motor modification among task conditions, a statistical analysis was performed on kinematics parameters by considering the average across the three repetitions of each task. Antero-posterior (AP), medio-lateral (ML), and vertical (V) components of the *^p^*^l^PT*_trk_* vector, the joint kinematics of the upper body, the lumbosacral joint (^RoM^Sacr) and the lower-limb joint kinematics (^RoM^Hip_L_, ^RoM^Hip_R_, ^RoM^Knee_L_, ^RoM^Knee_R_, ^RoM^Ankle_L_, and ^RoM^Ankle_R_) values were considered in the statistical analysis. A 2 × 3 two-way repeated measures analysis of variance (ANOVA) was performed separately on the LL and RL tasks, with main effects: *Posture* and *Weight* as within-subject factors. More specifically, *Posture* presented two levels related to the two postural patterns, correct and incorrect, while *Weight* presented three levels of 1, 2, and 5 kg. The significance level was set at 0.05 for all statistical tests, and the Greenhouse–Geisser correction was adopted when the Mauchly’s test was significant and the assumption of sphericity was violated. A Bonferroni test was used for post-hoc analysis. If the interaction effect of *Posture* × *Weight* was significant, we broke down the interactions by comparing the LL and RL tasks in the correct and the incorrect postures with paired t-tests at each level of *Weight*.

### 2.5. Machine Learning Classifier

Posture classification was obtained through a support vector machine (SVM), which guarantees a high classification performance even with a small dataset, as shown in a study by Zhang et al. [3,47]. The SVM classifier training and testing dataset consisted of kinematic parameters that were estimated through the BM. According to our kinematic parameters, the ten features consisted of the excursion range of the displacement of the *^p^*^l^PT*_trk_* vector in the AP, ML, and V directions; the ranges of motion of lumbosacral joint and the lower limb joints that were obtained by the three repetitions of each task that was executed by each subject. We tested: (i) a dataset with all ten features, which was named the trunk–lower limb (T-Ll) dataset and considered all measured kinematic parameters of both the trunk and lower limbs, and (ii) a smaller dataset with only four features that was named the upper body (UpB) dataset and considered the kinematic parameters related to the upper body, *^pl^*PT*_trk_,* and ^RoM^Sacr. Furthermore, these two datasets were both divided into six sub-datasets by considering the two postural patterns, i.e., correct and incorrect, and each load, i.e., 1, 2, and 5 kg. The machine-learning algorithm and the analysis of its performance were obtained by using the MATLAB (v.2018, Math Works, Natick, MA) software.

Before training and testing the SVM classifier, the features were normalized, by subtracting the mean and dividing by the standard deviation of each feature. A leave-one-out cross-validation was adopted to evaluate the performance of the classifier. As performance measurements, we considered:

- Accuracy (Acc)

(6) $$\left(\frac{TP+TN}{TP+FP+TN+FN}\right)\times 100\%$$

- Specificity (True Negative Rate—TNR)

(7) $$\left(\frac{TN}{TN+FP}\right)\times 100\%$$

- Sensitivity/Recall (True Positive Rate—TPR)

(8) $$\left(\frac{TP}{TP+FN}\right)\times 100\%$$

- Precision (Positive Predictive Value—PPV)

(9) $$\left(\frac{TP}{TP+FP}\right)\times 100\%$$

The true positive (TP) is the number of true positives, i.e., the SVM identifies the LL task or the RL task that was labelled as the task that was performed in the incorrect posture; true negative (TN) is the number of true negatives, i.e., SVM identifies the LL task or the RL task as that which was labelled as the task that was performed in the correct posture; false positive (FP) is the false identification of the incorrect posture; and false negative (FN) is the false identification of the correct posture. TNR quantifies how classifier could avoid false detection in order to correctly discriminate the two postures. TPR defines the ability of the SVM to recognize the MMH tasks that were executed in the incorrect posture. PPV allows for the identification of how many tasks were executed in the incorrect posture were correctly classified. We used those performance measurements to test SVMs with four different kernel functions: linear, polynomial (quadratic and cubic), and Gaussian.

## 3. Results

### 3.1. Results of the Statistical Analysis

**Figure 2:**
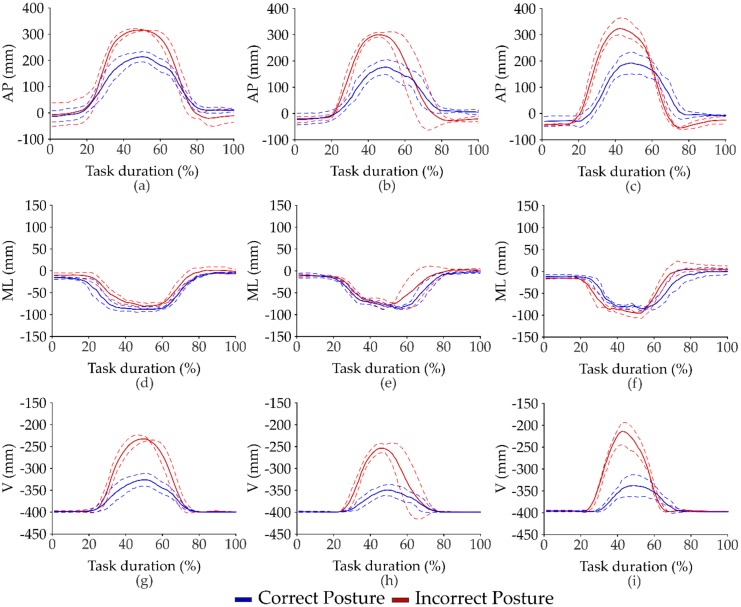
Mean (solid line) and standard deviation (dashed line) values of the range excursion of the displacement of the *^pl^*PT*_trk_* vector in the AP, ML and V directions with respect to the task duration of a subject performing the CLL and ILL tasks with 1 kg (**a**,**d**,**g**), 2kg (**b**,**e**,**h**) and 5 kg (**c**,**f**,**i**) loads.

**Figure 3:**
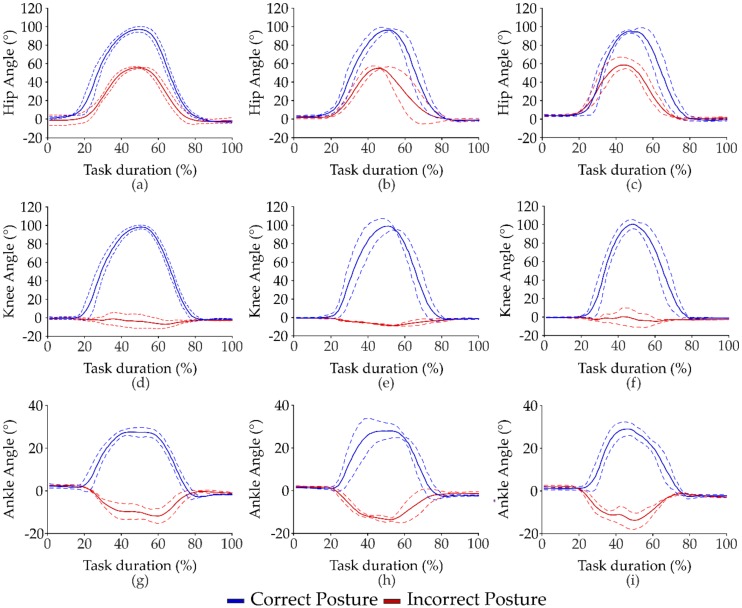
Mean (solid line) and standard deviation (dashed line) values of the range excursion of the right lower limb joint angles in the sagittal plane with respect to the task duration of a subject performing the CLL and ILL tasks with 1 kg (**a**,**d**,**g**), 2kg (**b**,**e**,**h**) and 5 kg (**c**,**f**,**i**) loads.

The mean and standard deviation values of all kinematic parameters, estimated through the BM over the three repetitions of tasks, and the results from statistical analysis are summarized in Table 1 for the LL task and in Table 2 for the RL task. No differences between the right and left side of the lower extremities were found. We only report mean and standard deviation values for the right lower limb joints in both Table 1 and Table 2. Figure 2 shows the mean and standard deviation values of the displacement of the *^p^*^l^PT*_trk_* vector in the AP, ML, and V directions of a subject performing the CLL and ILL tasks with the three loads. The displacement was normalized at task duration and is expressed as a percentage. Mean and standard deviation values of right lower limb joint angles in the sagittal plane in respect to the task duration of a subject performing the CLL and the ILL tasks with the three loads are reported in Figure 3. We set the angles of hip flexion as positive, knee flexion, and ankle dorsiflexion.

No significant interaction was found for all considered parameters in the LL task. Considering the LL task*,* statistical differences were found for all kinematic parameters in the main effect of posture (*p* < 0.01). In the main effect of weight, significant differences were found for the ML component of the *^p^*^l^PT*_trk_* vector (*p* < 0.01) and for the ^RoM^Hip (*p* < 0.01, for both sides). In regards to the ML component of the *^p^*^l^PT*_trk_* vector, *the* Bonferroni test showed differences between the 1 and 5 kg loads (*p* = 0.02) and between the 2 and 5 kg loads (*p* = 0.01)*_._*

Considering the range of motion of the right hip, differences were found between the 1 and 5 kg loads (*p* < 0.01), between the 2 and 5 kg loads (*p* < 0.01), and between the 1 and 2 kg loads (*p* < 0.01). As for the range of motion of the left hip, differences were found between the 1 and 5 kg loads (*p* < 0.01), between the 2 and 5 kg loads (*p* = 0.02), and between the 1 and 2 kg loads (*p* < 0.01).

In the RL task, a similar trend was observed in the main effect of posture. In fact, significant differences were found for all kinematic parameters (*p* < 0.01). In the main effect of weight, significant differences were found only for the ^RoM^Hip (*p* < 0.01, of both side). In regard the ^RoM^Hip, the Bonferroni test showed differences between the 1 and 5 kg loads (*p* < 0.01, of both sides) and between the 1 and 2 kg loads (*p* < 0.01, of both sides). A significant interaction for the ML component of the *^p^*^l^PT*_trk_* vector was found. Breaking down the two-way repeated measures ANOVA in three paired t-tests, statistical differences were found between the CRL task and the IRL task with the 2 kg load (*p* < 0.01) and the 5 kg load (*p* < 0.01).

### 3.2. Results of Machine Learning Classifier

In Table 3 and Table 4, the performance measurements of the classifier in terms of accuracy (Acc), TNR, TPR and PPV are reported for the LL task and the RL task, respectively. Regarding the SVM that was fed with all kinematic parameters, the accuracy rate reached 98.7% in the LL task and 99.4% in the RL task with the 1 kg load through the SVM linear kernel. In the LL task with a 2 kg load, the highest values of accuracy (99.4%) and TPR (100%) were found through the SVM quadratic kernel rather than in the other considered kernel functions, while the highest values of TNR and PPV (100%) were found through the SVM Gaussian kernel. Considering the 5 kg load, a 98.1% accuracy rate was reached for the LL task and a 98.7% accuracy rate was reached for the RL task through the SVM linear kernel. Regarding PPV, it reached its highest values (98.7%) in the LL task and (100%) in the RL task through the linear kernel. Furthermore, in the T-Ll dataset, good results of TNR were obtained for the LL and RL tasks with all three loads. Specifically, we obtained a TNR of 98.7% in the LL task and a TNR of 100% in the RL task through the SVM linear kernel with the highest load.

**Figure 4:**
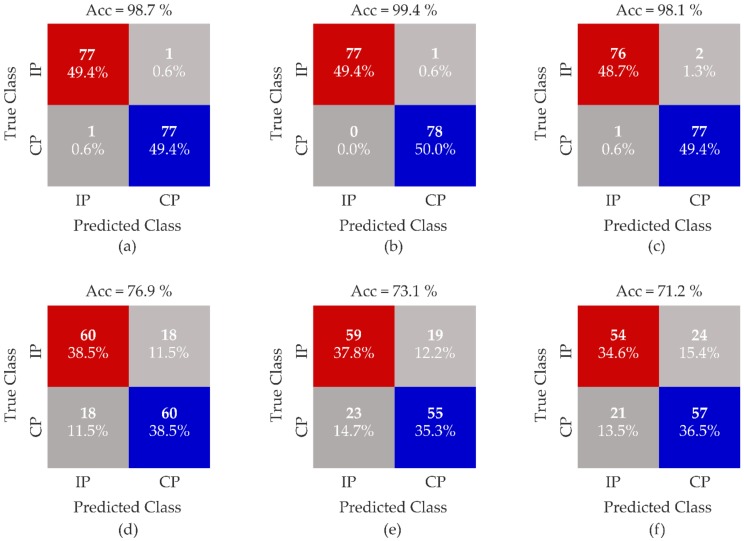
Confusion matrices for the T-Ll dataset for the LL task with the 1 kg linear kernel (**a**), 2 kg quadratic kernel (**b**) and 5 kg linear kernel (**c**) loads; and the UpB dataset for the LL task with the 1 kg quadratic kernel (**d**), 2 kg Gaussian kernel (**e**) and 5 kg linear kernel (**f**). IP = incorrect Posture and CP = correct Posture.

In Figure 4, the confusion matrices of the classifiers related to the two datasets are reported when considering only the highest values of accuracy in the LL task with the three loads. Focusing on the T-Ll dataset, the highest accuracy was reached through the SVM linear kernel for the 1 kg load with a processing time of 3.43 s, for the 2 kg load through the SVM quadratic (1.62 s) and Gaussian kernel (1.52 s), and for the 5 kg load through the SVM linear kernel (1.78 s). Regarding the UpB dataset, the highest accuracy was reached through the SVM quadratic kernel for the 1 kg load (780.40 s), for the 2 kg load through the SVM Gaussian kernel (1.88 s), and for the 5 kg load through the SVM linear kernel (82.11 s).

**Figure 5:**
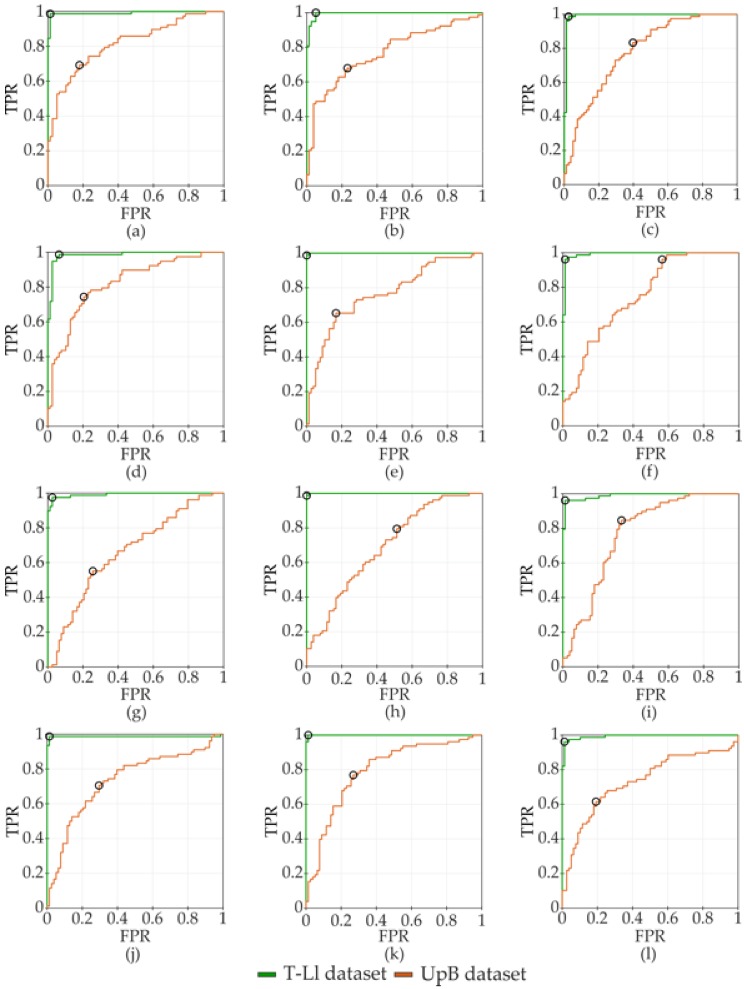
Receiver operating characteristic (ROC) curves of the TL-l dataset and the UpB dataset of the LL task with: linear kernel 1 kg (**a**), 2kg (**b**), and 5 kg (**c**) loads; quadratic kernel 1 kg (**d**), 2 kg (**e**), and 5 kg (**f**) loads; cubic kernel 1 kg (**g**), 2 kg (**h**), and 5 kg (**i**) loads; and Gaussian kernel 1 kg (**j**), 2 kg (**k**) and 5 kg (**l**) loads. The black circle represents the optimal ROC operating point.

In Figure 5, the receiver operating characteristic (ROC) curves of SVM related to the TL-l dataset and the UpB dataset are represented only for the LL task in order to compare the performance of the SVM between the considered kernel function.

## 4. Discussion

The kinematic parameters, obtained through the biomechanical model, allowed for the unveiling of differences between the correct and the incorrect postures in lifting/releasing load tasks. Bending the trunk and keeping the trunk flexed for a long time could favor the onset of spinal disorders during a lifting load task [4,23,39]. As shown in Figure 1, the AP, ML and V components of the *^pl^*PT*_trk_* vector reached high mean values while a subject lifted load with the trunk completely bended. These kinematic parameters, considered to detect the role of the upper body in the MMH tasks, allow for the observation of differences between the two postural patterns with the three loads, as demonstrated by statistical analysis. In Figure 2, the AP and the V components of the *^pl^*PT*_trk_* vector show a comparable duration for the descent phase, i.e., moving to reach the load, and the ascent phase, i.e., moving to lift the load, in the correct posture. Considering the LL task for the 1 kg load, we did not observe differences for the AP and the V components during the two phases in both postures. Our hypothesis is that the 1 kg load was easier to handle with respect to the other considered loads for all the subjects of the study, showing a minimal effect on their movement.

In the incorrect posture, the ascent phase appeared to have been performed faster than the descent phase in the LL task, especially for the 2 and 5 kg loads (see Figure 1), as can be seen by looking at the AP and the V components. On the contrary, we observed no modifications of the ML component of the *^pl^*PT*_trk_* vector during the ascent and the descent phase of the LL task (see Figure 2).

In Figure 3, a wide excursion of the hip angle is observable during the LL task in the correct posture, which is considerably different from the incorrect one, as demonstrated by statistical results. In Figure 2, a reduction of the range of motion of the knee and ankle joints in the incorrect posture during the LL task is observable with respect to the correct one. These results of the range of motion of the knee and ankle joints are closely related to the motion pattern of the two postures (Table 1 and Table 2), as we expected.

The study of Valero et al*,* highlighted how workers tend to increase productivity without taking care of healthy postures, dramatically favoring risk of WMSDs [2]. They noticed that a safe posture during working tasks was maintained by workers with more experience in their profession compared to their less experienced colleagues. No correlation was found between experience and posture, while an increase of productivity was correlated with a reduced control of the body posture of the workers [2]. These observations underline the need for relying on measurements of posture parameters in the workplace. Our biomechanical model and our wearable setup allowed for a reliable measurement of these parameters, quantifying important movement variables of the two considered postural patterns on the lower limbs, as highlighted in the results of our previous study [43]. By increasing the number of subjects, we obtained significance for all considered kinematic parameters in the main effect of posture. Thus, increasing the mobility of the lower extremities allows for the maintenance of a straight trunk, avoiding unsafe biomechanical stress on the spine and ensuring a better control in this MMH tasks.

In the main effect of weight, we found no significance for the AP and V components of the *^pl^*PT*_trk_* vector, and we could not quantify a dependency on the weight increase of load. Only for the ML component was a dependency on the weight observed, despite the results of a previous analysis on the kinematic parameters of the trunk [43]. As shown in Table 1, we observed small differences in the CLL and CRL tasks, while we found an increase of the mean value related to the increase of load in the ILL and the IRL tasks. In our experimental protocol, the LL and RL tasks were executed symmetrically, so we did not expect high excursion in the ML direction of the trunk with respect to the AP and V directions. Despite the statistical significance, the variation of this parameter in the single trial of each task was not sufficient to discriminate between the two postural patterns. Furthermore, interesting results were found for the RoM of the hip joint, as demonstrated by the statistical analysis. According to the previous results on the hip joint [43], we could confirm the effects that were related to the weight increase of the load on this parameters (on both sides) for the two tasks. These results allow us to reaffirm the necessity to include lower limb segments in the ergonomic evaluation of MMH tasks. Regarding the knee joint, high standard deviation values were obtained in the ILL and the IRL tasks. This bias may have been due either to the sample size or to the different strategies that were adopted by each subject in each condition, i.e., postural patterns or loads. In future developments, we will further investigate this aspect.

According to our results, some subjects could feel safer being close to the load before lifting with the correct posture, while others adopted the strategy that included bending the knees. Furthermore, they could not limit the flexion of their trunk, as demonstrated by the results. Hwang et al. estimated joint angles when the lumbar lordosis appeared during the lifting task [33]. They found significant differences of the lumbosacral joint with respect to the weight increase and a correlation between lower limb joints and the lumbosacral joint during the squat lifting and stoop lifting techniques. In the case of the 5 kg load, we can consider our results similar to those of Hwang et al. for all joint angles [33].

The impossibility of setting a precise and reliable threshold for each considered parameter called for a machine learning solution to gather more complete information coming from all body segments. We tested the SVM with the two datasets: TL-l dataset, which was obtained through data that were collected with the eight IMUs, and the UpB dataset, which was composed of data that were collected through two sensors that were placed on pelvis and trunk body segments. The UpB dataset could not represent an effective solution for posture recognition, especially in real time application, considering the time processing and measurements of performance of the considered machine-learning algorithm. The SVM classifier showed a high accuracy rate in the LL and RL tasks with the three loads through T-Ll dataset. Both TPR and TNR values were high, demonstrating a balanced performance of the classifier in distinguishing the two postures through the T-Ll dataset. Specifically, the highest accuracy was found through the SVM polynomial and Gaussian kernel in the LL task and through the SVM linear and polynomial kernel in the RL task with the 2 kg load. In the RL task with the 5 kg load, we obtained a 100% TPR value and a 97.4% TNR value (Table 4). In the study of Alwasel et al., a high accuracy was reached in distinguishing between safe and unsafe postures assumed by expert and inexpert masonry workers [52]. They implemented an SVM that was fed with inertial data of the whole kinematic chain. Among the three considered SVM kernel functions, linear and polynomial ones were found to perform classification with high accuracy (92%), similarly to our results. Regarding the Gaussian kernel function, they did not find a high value of accuracy (89%) [52]. Conversely, we obtained a high accuracy with such a kernel, especially in the LL task with the 2 kg load.

Considering the reduced UpB dataset, the SVM Gaussian kernel was found to perform classification with good accuracy for the LL and RL tasks with all loads. As shown in Figure 3, even if we found a good rate of accuracy in the LL task, the FP assumed values that increased with the weight of loads, especially between the 1 and 2 kg loads. This result confirmed that the use of features related to a body segment, e.g., the trunk, does not allow for the correct identification of the two postural patterns. The weakness of this dataset could be in one feature of the trunk, the excursion range of the ML displacement of the *^pl^*PT*_trk_* vector. In our experimental protocol, the LL and RL tasks were executed symmetrically, so we did not expect high alterations of these parameters with respect to the other kinematic parameters of the trunk body segment that were estimated through the BM. The assessment of the TPR and the TNR guaranteed a reliable analysis of the performance of the considered classifier. The misclassification of an unsafe posture could not help to prevent WMSDs, exposing workers to biomechanical risk. On the contrary, the misclassification of the correct posture does not expose the user to any risk, even if it compromises the reliability of the classifier, which in turn could lead to the abandoning of the use of the solution. Thus, considering only kinematic parameters that are related to trunk segment does not allow for a reliable performance of the algorithm (see Figure 4 and Figure 5).

Chen et al. trained an SVM algorithm with predefined posture classes to let the model discriminate postures in a set of activities. Considering different combinations of training parameters to obtain a high value of accuracy, they could not correctly classify squatting and stooping, even if they considered a dataset representing the whole kinematic chain [7]. The discrimination of unsafe biomechanical loads was better performed by the T-Ll dataset than the UpB dataset, where the obtained rate of accuracy might not have been enough to identify a non-ergonomic working posture. As shown in Figure 4c, we obtained an FP value of two, so the classifier misclassified the lifting load task that was executed in the incorrect posture for one repetition of the task for two subjects. Regarding the FN value, the classifier misclassified the lifting load task that was performed in the correct posture for one repetition of the task of one subject. In Figure 4f, the FN value is shown to be higher than the FN value in Figure 4c. The FN value that was equal to twenty-one shows that the classifier misclassified the correct posture with the incorrect posture for those subjects who may not have varied the motion of the trunk body segment in the two postural patterns during the lifting load task. These results confirm that a small dataset, with features related to one body segment, does not give any effective information on the postural patterns and the motion phases of tasks. Regarding the processing time of the SVM algorithm with the best accuracy (see Figure 4), an effective classification was obtained through the SVM quadratic kernel for the TL-l dataset and through the Gaussian kernel for the UpB dataset. Nevertheless, when considering the level of accuracy and the high values of FP and FN, a small dataset is not an effective choice (see Figure 4 and Figure 5). Furthermore, when considering the TL-l dataset, the processing time result was lower than the result that was obtained in Alwasel et al. study [52].

The main limitations of our study are related to the reduction of the working factors for the MMH tasks in our experimental protocol, which did not examine other important working factors. In the future development of the methodology, we will also explore uncomfortable grip and asymmetrical lifting to quantify sensitivity to load variation of the medio-lateral displacement of the trunk, the frequency and repetition of the tasks to assess the effect of fatigue, and loads greater than 5 kg. A further limitation is related to the small size of the sample, which was composed by unexperienced volunteers. In future developments, we will increase the number of participants by recruiting experienced workers.

## 5. Conclusions

Measurements of kinematic parameters that are related to the trunk body segment and lower limb joints and obtained through our biomechanical model allowed us to distinguish between a safe and an unsafe posture. Interesting results on alterations of hip joint were observed when considering low loads, as highlighted by statistical analysis. Moreover, the SVM classifier reached high rates of accuracy, specificity, and sensitivity in discriminating the two postural patterns through all considered kinematic parameters. On the contrary, the outcome of the SVM classifiers that were fed with kinematic parameters related only to the trunk does not allow for a satisfactory analysis of risk. The success of a biomechanical evaluation of risks in work environment, preventing non-fatal occupational injuries, depends on a full motion analysis of a worker’s performance, i.e., including the contribution of lower limb kinematics. To reduce overexertion in MMH tasks, mitigating the occurrence of back spasms and sprains, the design of a real-time posture-monitoring tool, combined with our wearable system and our biomechanical model, will be a powerful solution in the workplace.

## Figures and Tables

**Table 1:** Mean, standard deviation, and *p-*values of the main effects *Posture* and *Weight* of a 2 x 3 two-way repeated measures ANOVA for the three components of the *^p^*^l^PT*_trk_* vector (the displacement in the 3D space between the pelvis and trunk body segments); the range of motion of the lumbosacral joint and the right lower limb joints estimated through the biomechanical model (BM) in the correct lifting loads (CLL) and incorrect lifting loads (ILL) tasks. * marks significant differences.

		CLL Task			ILL Task			*p*-Value	
		1 kg	2 kg	5 kg	1 kg	2 kg	5 kg	*Posture*	*Weight*
***^p^*^l^PT*_trk_***	AP (mm)	222 ± 68	215 ± 74	215 ± 71	298 ± 59	288 ± 62	284 ± 76	<0.01 *	0.21
	ML (mm)	41 ± 19	41 ± 21	46 ± 20	48 ± 24	51 ± 25	58 ± 30	<0.01 *	<0.01 *
	V (mm)	80 ± 50	76 ± 55	75 ± 49	151 ± 54	137 ± 54	140 ± 62	<0.01 *	0.27
**RoM**	Sacr (°)	36.7 ± 12.4	35.7 ± 14.2	35.4 ± 13.1	52.5 ± 10.0	49.9 ± 11.1	49.6 ± 13.4	<0.01 *	0.17
	Hip (°)	99.4 ± 10.7	102.1 ± 11.3	104.5 ± 13.2	75.3 ± 15.2	77.6 ± 15.6	80.0 ± 16.5	<0.01 *	<0.01 *
	Knee (°)	102.1 ± 16.8	104.4 ± 17.8	106.1 ± 17.4	29.6 ± 20.8	28.1 ± 19.7	30.5 ± 18.6	<0.01 *	0.08
	Ankle (°)	27.6 ± 7.9	27.8 ± 7.5	28.1 ± 7.4	12.8 ± 4.9	12.8 ± 5.2	12.8 ± 4.6	<0.01 *	0.80

**Table 2:** Mean, standard deviation, and *p-*values of the main effects *Posture* and *Weight* of a 2 × 3 two-way repeated measures ANOVA for the three components of the *^p^*^l^PT*_trk_* vector (the displacement in the 3D space between the pelvis and trunk body segments); the range of motion of the lumbosacral joint and the right lower limb joints estimated through the biomechanical model (BM) in the correct releasing loads (CRL) and incorrect releasing loads (IRL) tasks.* marks significant differences.

		CRL Task			IRL Task			*p*-Value	
		1 kg	2 kg	5 kg	1 kg	2 kg	5 kg	*Posture*	*Weight*
***^p^*^l^PT*_trk_***	AP (mm)	234 ± 73	231 ± 75	226 ± 75	304 ± 57	295 ± 61	287 ± 75	<0.01 *	0.14
	ML (mm)	48 ± 21	47 ± 23	49 ± 20	52 ± 25	57 ± 26	61 ± 29	<0.01 *	0.11
	V (mm)	89 ± 60	86 ± 61	85 ± 53	162 ± 58	143 ± 57	146 ± 63	<0.01 *	0.26
**RoM**	Sacr (°)	39.3 ± 14.2	38.5 ± 14.8	37.5 ± 14.1	55.0 ± 9.9	51.6 ± 11.3	50.6 ± 12.9	<0.01 *	0.09
	Hip (°)	100.4 ± 11.5	103.4 ± 11.0	105.7 ± 11.5	75.0 ± 16.4	77.1 ± 16.6	78.0 ± 16.2	<0.01 *	<0.01 *
	Knee (°)	104.1 ± 17.1	106.8 ± 18.4	108.9 ± 17.9	28.2 ± 19.3	27.9 ± 19.5	27.4 ± 17.5	<0.01 *	0.27
	Ankle (°)	26.8 ± 7.7	26.8 ± 7.7	26.2 ± 7.2	12.4 ± 4.1	11.9 ± 5.1	12.0 ± 4.2	<0.01 *	0.44

**Table 3:** Overall accuracy (Acc), specificity (true negative rate (TNR)), sensitivity/recall (true positive rate (TPR)) and precision (positive predictive value (PPV)) values of the posture recognition when using the four different kernel functions considering the trunk–lower limb (T-Ll) and upper body (UpB) datasets in the LL task.

		LL Task											
		1 kg				2 kg				5 kg			
	**SVM kernels**	Acc	TNR	TPR	PPV	Acc	TNR	TPR	PPV	Acc	TNR	TPR	PPV
**T-Ll**	*Linear*	98.7	98.7	98.7	98.7	94.9	94.9	94.9	94.9	98.1	98.7	97.4	98.7
	*Quadratic*	95.5	96.2	94.9	96.1	99.4	98.7	100	98.7	97.4	96.2	98.7	96.3
	*Cubic*	96.8	97.4	96.2	97.4	98.7	98.7	98.7	98.7	96.8	96.2	97.4	96.2
	*Gaussian*	98.1	98.7	97.4	98.7	99.4	100	98.7	100	97.4	96.2	98.7	96.3
**UpB**	*Linear*	73.7	70.5	76.9	72.3	72.4	67.9	76.9	70.6	71.2	73.1	69.2	72.0
	*Quadratic*	76.9	76.9	76.9	76.9	71.8	70.5	73.1	71.3	67.3	67.9	66.7	67.5
	*Cubic*	62.2	56.4	67.9	60.9	62.8	55.1	70.5	61.1	70.5	69.2	71.8	70.0
	*Gaussian*	69.2	66.7	71.8	68.3	73.1	70.5	75.6	72.0	69.2	69.2	69.2	69.2

**Table 4:** Overall accuracy (Acc), specificity (true negative rate (TNR)), sensitivity/recall (true positive rate (TPR)) and precision (positive predictive value (PPV)) values of the posture recognition when using the four different kernel functions considering the trunk–lower limb (T-Ll) and upper body (UpB) datasets in the RL task.

		RL Task											
		1 kg				2 kg				5 kg			
	**SVM kernels**	Acc	TNR	TPR	PPV	Acc	TNR	TPR	PPV	Acc	TNR	TPR	PPV
**T-Ll**	*Linear*	99.4	98.7	100	98.7	99.4	98.7	100	98.7	98.7	100	97.4	100
	*Quadratic*	98.1	98.7	97.4	98.7	99.4	98.7	100	98.7	94.9	93.6	96.2	93.8
	*Cubic*	97.4	98.7	96.2	98.7	99.4	98.7	100	98.7	95.5	93.6	97.4	93.8
	*Gaussian*	98.1	97.4	98.7	97.5	98.7	97.4	100	97.5	98.1	98.7	97.4	98.7
**UpB**	*Linear*	72.4	67.9	76.9	70.6	67.9	69.2	66.7	68.4	70.5	82.1	59.0	76.7
	*Quadratic*	71.2	62.8	79.5	68.1	74.4	70.5	78.2	72.6	76.3	73.1	79.5	74.7
	*Cubic*	78.8	83.3	74.4	81.7	67.9	69.2	66.7	68.4	68.6	59.0	78.2	65.6
	*Gaussian*	83.3	78.2	88.5	80.2	76.9	80.8	73.1	79.2	71.2	65.4	76.9	69.0

## References

[1] Antwi-Afari M.F., Li H., Edwards D.J., Pärn E.A., Seo J., Wong A.Y.L. (2017). Biomechanical analysis of risk factors for work-related musculoskeletal disorders during repetitive lifting task in construction workers. Autom. Constr..

[2] Valero E., Sivanathan A., Bosché F., Abdel-Wahab M. (2017). Analysis of construction trade worker body motions using a wearable and wireless motion sensor network. Autom. Constr..

[3] Ray S.J., Teizer J. (2012). Real-time construction worker posture analysis for ergonomics training. Adv. Eng. Informatics.

[4] Yan X., Li H., Li A.R., Zhang H. (2017). Wearable IMU-based real-time motion warning system for construction workers’ musculoskeletal disorders prevention. Autom. Constr..

[5] Reid C.R., McCauley Bush P., Karwowski W., Durrani S.K. (2010). Occupational postural activity and lower extremity discomfort: A review. Int. J. Ind. Ergon..

[6] Pope M.H., Goh K.L., Magnusson M.L. (2002). Spine Ergonomics. Annu. Rev. Biomed. Eng..

[7] Chen J., Qiu J., Ahn C. (2017). Construction worker’s awkward posture recognition through supervised motion tensor decomposition. Autom. Constr..

[8] Yan X., Li H., Wang C., Seo J.O., Zhang H., Wang H. (2017). Development of ergonomic posture recognition technique based on 2D ordinary camera for construction hazard prevention through view-invariant features in 2D skeleton motion. Adv. Eng. Inform..

[9] Nath N.D., Akhavian R., Behzadan A.H. (2017). Ergonomic analysis of construction worker’s body postures using wearable mobile sensors. Appl. Ergon..

[10] Chen J., Ahn C.R., Han S. (2014). Detecting the Hazards of Lifting and Carrying in Construction through a Coupled 3D Sensing and IMUs Sensing System. Comput. Civ. Build. Eng..

[11] Jebelli H., Ahn C.R., Stentz T.L. (2016). Fall risk analysis of construction workers using inertial measurement units: Validating the usefulness of the postural stability metrics in construction. Saf. Sci..

[12] Spielholz P., Davis G., Griffith J. (2006). Physical risk factors and controls for musculoskeletal disorders in construction trades. J. Constr. Eng. Manag..

[13] Wang D., Dai F., Ning X. (2015). Risk assessment of work-related musculoskeletal disorders in construction: State-of-the-art review. J. Constr. Eng. Manag..

[14] Umer W., Li H., Szeto G.P.Y., Wong A.Y.L. (2017). Identification of Biomechanical Risk Factors for the Development of Lower-Back Disorders during Manual Rebar Tying. J. Constr. Eng. Manag..

[15] Cho Y.K., Kim K., Ma S., Ueda J. A robotic wearable exoskeleton for construction worker’s safety and health. Proceedings of the ASCE Construction Research Congress 2018.

[16] CDC—NIOSH Publications and Products Ergonomic Guidelines for Manual Material Handling (2007-131). https://www.cdc.gov/niosh/docs/2007-131/default.html.

[17] Marras W.S., Parakkat J., Chany A.M., Yang G., Burr D., Lavender S.A. (2006). Spine loading as a function of lift frequency, exposure duration, and work experience. Clin. Biomech..

[18] Ning X., Zhou J., Dai B., Jaridi M. (2014). The assessment of material handling strategies in dealing with sudden loading: The effects of load handling position on trunk biomechanics. Appl. Ergon..

[19] Kingma I., Baten C.T.M., Dolan P., Toussaint H.M., Van Dieën J.H., De Looze M.P., Adams M.A. (2001). Lumbar loading during lifting: A comparative study of three measurement techniques. J. Electromyogr. Kinesiol..

[20] Leskinen T.P.J., Stålhammar H.R., Kuorinka I.A.A., Troup J.D.G. (1983). A dynamic analysis of spinal compression with different lifting techniques. Ergonomics.

[21] Hoozemans M.J.M., Kingma I., de Vries W., van Dieën J. (2008). Effect of lifting height and load mass on low back loading. Ergonomics.

[22] Harari Y., Riemer R., Bechar A. (2019). Differences in spinal moments, kinematics and pace during single-task and combined manual material handling jobs. Appl. Ergon..

[23] Application Manual for the Revised NIOSH Lifting Equation. https://www.cdc.gov/niosh/docs/94-110/pdfs/94-110.pdf?id=10.26616/NIOSHPUB94110.

[24] NIOSH (2014). Observation-based posture assessment: Review of current practice and recommendations for improvement. Anim. Genet..

[25] Straker L. (2003). Evidence to support using squat, semi-squat and stoop techniques to lift low-lying objects. Int. J. Ind. Ergon..

[26] Van Dieen J.H., Van Hoozemans M.J.M., Van Toussaint H.M. (2000). A Review of Biomechanical Studies on Stoop and Squat Lifting. Proc. Hum. Factors Ergon. Soc. Annu. Meet..

[27] Bazrgari B., Shirazi-Adl A., Arjmand N. (2007). Analysis of squat and stoop dynamic liftings: Muscle forces and internal spinal loads. Eur. Spine J..

[28] McAtamney L., Nigel Corlett E. (1993). RULA: A survey method for the investigation of work-related upper limb disorders. Appl. Ergon..

[29] Hignett S., McAtamney L. (2000). Rapid Entire Body Assessment (REBA). Appl. Ergon..

[30] Kee D., Karwowski W. (2007). A Comparison of Three Observational Techniques for Assessing Postural Loads in Industry. Int. J. Occup. Saf. Ergon..

[31] David G.C. (2005). Ergonomic methods for assessing exposure to risk factors for work-related musculoskeletal disorders. Occup. Med. (Chic. Ill)..

[32] Buchholz B., Paquet V., Punnett L., Lee D., Moir S. (1996). PATH: A work sampling-based approach to ergonomic job analysis for construction and other non-repetitive work. Appl. Ergon..

[33] Hwang S., Kim Y., Kim Y. (2009). Lower extremity joint kinetics and lumbar curvature during squat and stoop lifting. BMC Musculoskelet. Disord..

[34] Schelldorfer S., Ernst M.J., Rast F.M., Bauer C.M., Meichtry A., Kool J. (2015). Low back pain and postural control, effects of task difficulty on centre of pressure and spinal kinematics. Gait Posture.

[35] Kollmitzer J., Oddsson L., Ebenbichler G.R., Giphart J.E., DeLuca C.J. (2002). Postural control during lifting. J. Biomech..

[36] Liu J., Zhang X., Lockhart T.E. (2012). Fall Risk Assessments Based on Postural and Dynamic Stability Using Inertial Measurement Unit. Saf. Health Work.

[37] Faber G.S., Chang C.C., Kingma I., Dennerlein J.T., van Dieën J.H. (2016). Estimating 3D L5/S1 moments and ground reaction forces during trunk bending using a full-body ambulatory inertial motion capture system. J. Biomech..

[38] Gholipour A., Arjmand N. (2016). Artificial neural networks to predict 3D spinal posture in reaching and lifting activities; Applications in biomechanical models. J. Biomech..

[39] Yan X., Li H., Zhang H., Rose T.M. (2018). Personalized method for self-management of trunk postural ergonomic hazards in construction rebar ironwork. Adv. Eng. Inform..

[40] Mileti I., Taborri J., Rossi S., Del Prete Z., Paoloni M., Suppa A., Palermo E. Measuring age-related differences in kinematic postural strategies under yaw perturbation. Proceedings of the MeMeA 2018—2018 IEEE International Symposium on Medical Measurements and Applications (MeMeA).

[41] Erra C., Mileti I., Germanotta M., Petracca M., Imbimbo I., De Biase A., Rossi S., Ricciardi D., Pacilli A., Di Sipio E. (2019). Immediate effects of rhythmic auditory stimulation on gait kinematics in Parkinson’s disease ON/OFF medication. Clin. Neurophysiol..

[42] Taborri J., Palermo E., Rossi S. (2019). Automatic detection of faults in race walking: A comparative analysis of machine-learning algorithms fed with inertial sensor data. Sensors (Switzerland).

[43] Conforti I., Mileti I., Del Prete Z., Palermo E. Assessing ergonomics and biomechanical risk in manual handling of loads through a wearable system. Proceedings of the 2019 IEEE International Workshop on Metrology for Industry 4.0 and IoT, MetroInd 4.0 and IoT 2019.

[44] Begg R., Kamruzzaman J. (2005). A machine learning approach for automated recognition of movement patterns using basic, kinetic and kinematic gait data. J. Biomech..

[45] Attal F., Mohammed S., Dedabrishvili M., Chamroukhi F., Oukhellou L., Amirat Y. (2015). Physical human activity recognition using wearable sensors. Sensors (Switzerland).

[46] Luštrek M., Kaluža B. (2009). Fall detection and activity recognition with machine learning. Informatica.

[47] Zhang J., Lockhart T.E., Soangra R. (2014). Classifying lower extremity muscle fatigue during walking using machine learning and inertial sensors. Ann. Biomed. Eng..

[48] Mileti I., Germanotta M., Di Sipio E., Imbimbo I., Pacilli A., Erra C., Petracca M., Rossi S., Del Prete Z., Bentivoglio A.R. (2018). Measuring gait quality in Parkinson’s disease through real-time gait phase recognition. Sensors (Switzerland).

[49] Suárez Sánchez A., Riesgo Fernández P., Sánchez Lasheras F., De Cos Juez F.J., García Nieto P.J. (2011). Prediction of work-related accidents according to working conditions using support vector machines. Appl. Math. Comput..

[50] Sun Y., Huang R., Zheng J., Dong D., Chen X., Bai L., Ge W. (2019). Design and speed-adaptive control of a powered geared five-bar prosthetic knee using bp neural network gait recognition. Sensors (Switzerland).

[51] Antwi-Afari M.F., Li H., Seo J.O., Wong A.Y.L. Automated Recognition of Construction Workers’ Activities for Productivity Measurement Using Wearable Insole Pressure System. Proceedings of the 2019 CIB World Building Congress.

[52] Alwasel A., Sabet A., Nahangi M., Haas C.T., Abdel-Rahman E. (2017). Identifying poses of safe and productive masons using machine learning. Autom. Constr..

[53] Ryu J., Seo J., Liu M., Lee S., Haas C.T. Action Recognition Using a Wristband-Type Activity Tracker: Case Study of Masonry Work. Proceedings of the Construction Research Congress 2016.

[54] Escamilla R.F. (2001). Squat Exercise. Med. Sci. Sports Exerc..

[55] Kritz M., Cronin J., Hume P., Zealand N., Zealand N., Science H. (2009). The Bodyweight Squat: A Movement Screen for the Squat Pattern. Natl. Strength Cond. Assoc..

[56] Palermo E., Rossi S., Marini F., Patanè F., Cappa P. (2014). Experimental evaluation of accuracy and repeatability of a novel body-to-sensor calibration procedure for inertial sensor-based gait analysis. Measurement.

[57] Pacilli A., Mileti I., Germanotta M., Di Sipio E., Imbimbo I., Aprile I., Padua L., Rossi S., Palermo E., Cappa P. A wearable setup for auditory cued gait analysis in patients with Parkinson’s Disease. Proceedings of the 2016 IEEE International Symposium on Medical Measurements and Applications (MeMeA).

